# Takotsubo Cardiomyopathy in the Setting of Myxedema Coma

**DOI:** 10.7759/cureus.32229

**Published:** 2022-12-05

**Authors:** Eric K Chan, Cameron Brown, Roudi Bachar, Antonio Smith, Sudarsana Srivangipuram

**Affiliations:** 1 Internal Medicine, Indiana University School of Medicine, Evansville, USA; 2 Internal Medicine, Good Samaritan Hospital, Vincennes, USA

**Keywords:** tsh, myxedema coma, takotsubo cardiomyopathy, thyroid hormone replacement, a case report

## Abstract

Myxedema coma and its treatment are potent physical stressors that likely predispose patients to develop Takotsubo cardiomyopathy (TCM). We postulate a multifactorial pathophysiology for TCM that includes a mechanism involving catecholamine-induced potentiation of cardiac adrenoreceptors during thyroid hormone replacement in the setting of severe hypothyroidism. Furthermore, TCM can be difficult to anticipate when presenting as a complication of another diagnosis. In this case report, we aim to improve awareness of TCM as a consequence of extreme hypothyroid states.

## Introduction

Myxedema coma is a medical emergency that results from uncontrolled hypothyroidism and carries a high mortality rate. Clinical manifestations include altered mental status, hypothermia, bradycardia, and coma that result from low triiodothyronine (T3) levels. Despite early diagnosis and treatment, the effects of myxedema coma place immense physical stress on the body which is crucial to recognize. Takotsubo cardiomyopathy (TCM) is characterized by apical wall systolic dysfunction that cannot be explained by perfusion defects and can be triggered by physical or emotional insults. In this case report, we present a case of TCM that developed as a complication of myxedema coma.

## Case presentation

**Figure 1:**
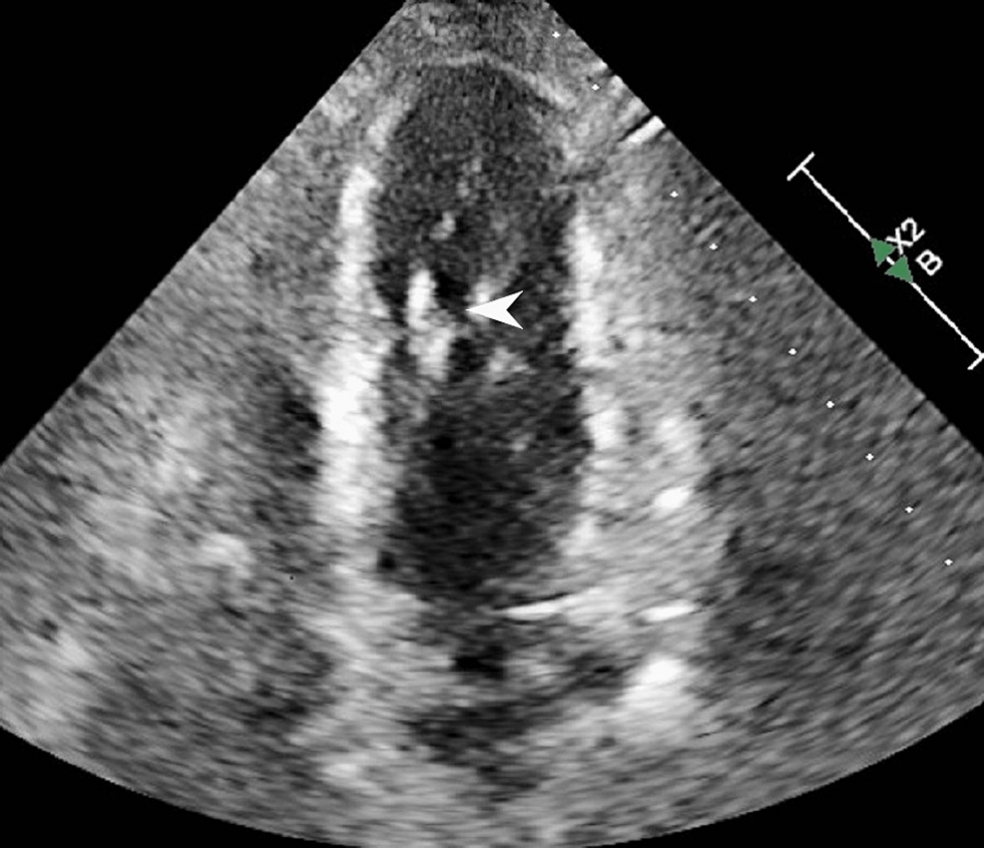
2D echocardiogram of the left ventricle in systole. Note the visualized close approximation of the endocardial border (arrow). 2D: Two-dimensional

**Figure 2:**
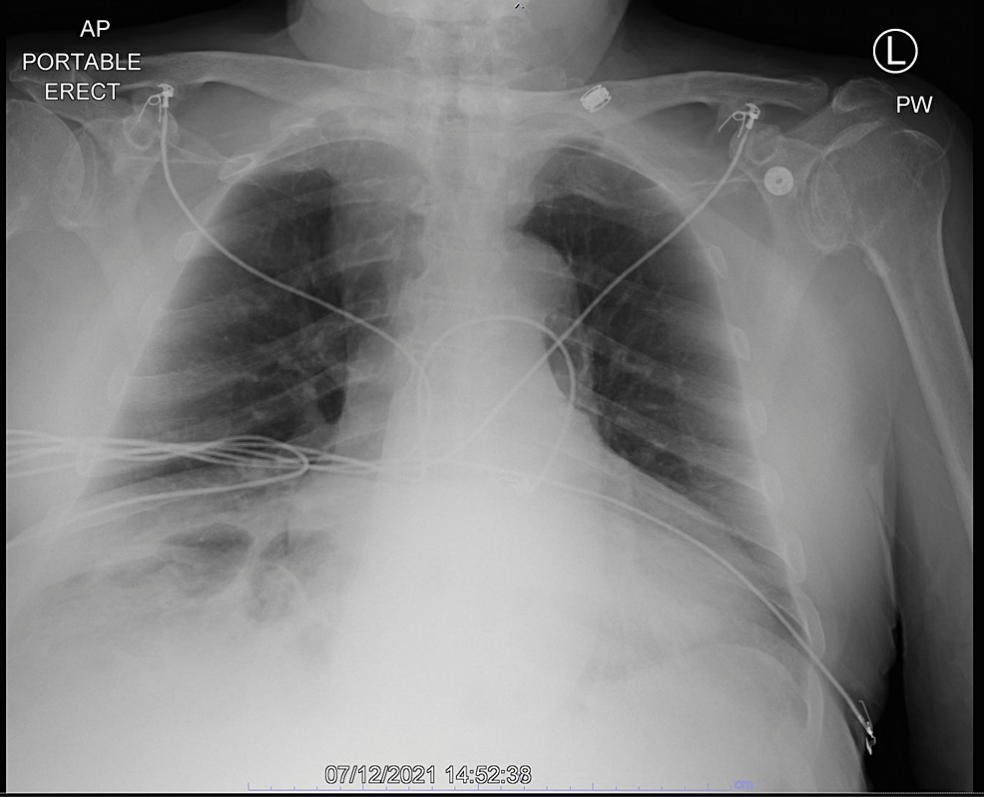
Initial chest X-ray. AP: Anteroposterior

The patient is a male in his 80s with known hypothyroidism, heart failure with preserved ejection fraction (EF), and chronic kidney disease and presented weakness, fatigue, dementia, dyspnea, constipation, cold intolerance, and falls at home. He reported non-adherence to his daily levothyroxine 150 mcg for an unknown amount of time. Initial vitals demonstrated bradycardia at 43 bpm with hypotension of 90/58 mmHg. Thyroid-stimulating hormone (TSH) was >150 mIU/L, and thyroxine (T4) was 0.02 ng/dL. Creatine phosphokinase was 1,367 U/L, aspartate aminotransferase was 51.0 U/L, and alanine aminotransferase was 31.0 U/L. An echocardiogram obtained on admission found grade 2 diastolic dysfunction, with otherwise normal left ventricle size and an EF of >55% (Figure 1). Portable chest X-ray found no diffuse airspace edema (Figure 2).

 

The patient was diagnosed with myxedema coma and treated with hydrocortisone 50 mg IV QID, levothyroxine 150 mcg PO QD, liothyronine 25 mcg PO QD that was increased to 50 mcg PO QD after two days, and gentle resuscitation with normal saline at 75mL/hr.

**Figure 3:**
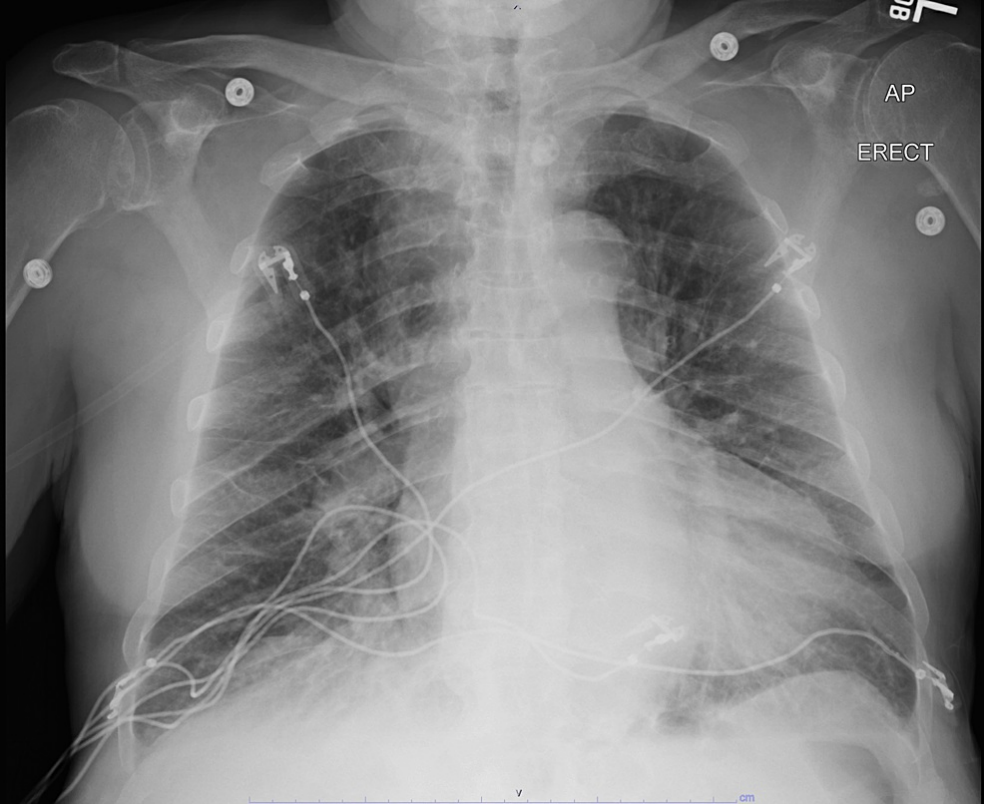
Chest X-ray after the development of pulmonary edema. AP: Anteroposterior

The patient’s condition rapidly improved following steroid and thyroid hormone supplementation with TSH decreasing to 10.47 mIU/L. However, on day seven of hospitalization, the patient developed flash pulmonary edema with associated chest pain, dyspnea, and pulmonary vascular congestion on imaging (Figure 3).

**Figure 4:**
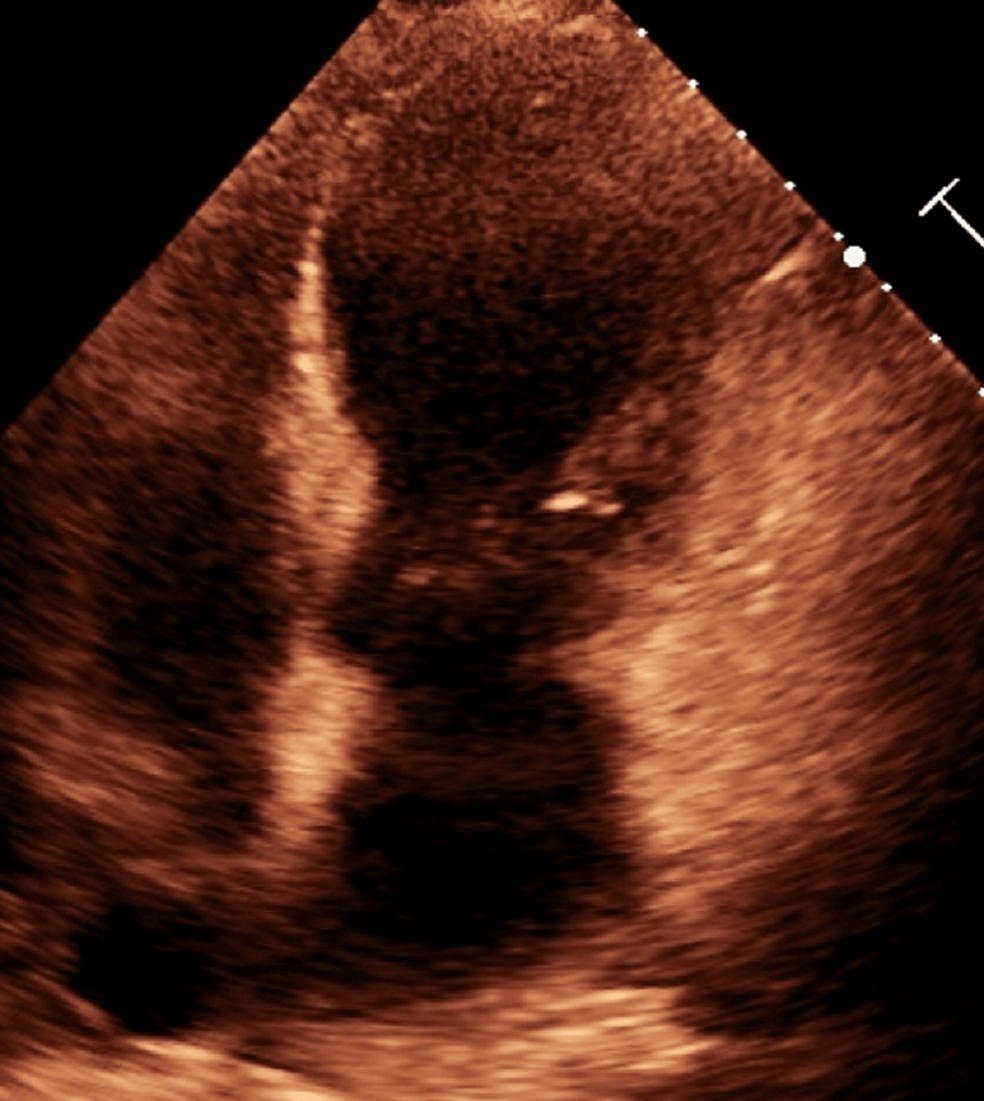
2D echocardiogram after the development of TCM. Note the characteristic apical ballooning. 2D: Two-dimensional TCM: Takotsubo cardiomyopathy

**Figure 5:**
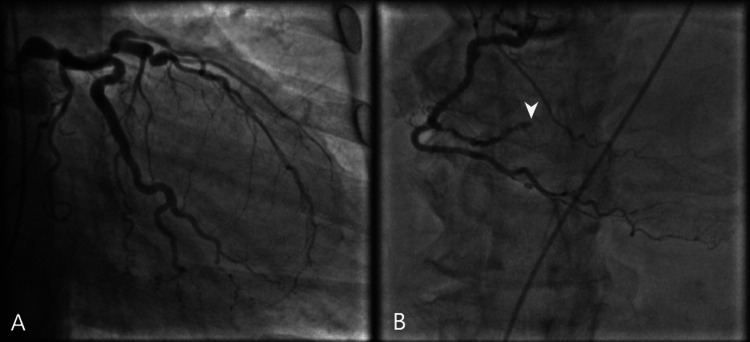
Coronary angiogram. Coronary angiogram following acute decompensation. (A) Imaging demonstrated non-obstructed vascular flow through the left main, left anterior descending, and left circumflex arteries. (B) Angiography of the right marginal branch of the right coronary artery found occlusion (arrow); however, this would not explain the hypokinesis observed in the left ventricle.

Cardiac evaluation demonstrated troponin elevation to 1789 pg/mL with no ST segment changes or new bundle branch block on EKG. Of note, QTc was elevated to 565 ms from 410 ms at admission. Repeat echocardiograms revealed a deteriorated EF of 15-20%, increased left ventricular cavity, and severe global hypokinesis (Figure 4). The patient was given 324 mg of aspirin and started on therapeutic dose Lovenox® before being transferred to an outside facility for cardiac evaluation. Subsequent cardiac catheterization found aneurysmal dilatation of the proximal-mid left anterior descending artery that reaches the apex with minimal luminal irregularities and diffuse disease of the RV marginal branch with subtotal terminal occlusion (Figure 5).

 

The lack of corresponding acute occlusive lesions in the distribution of his wall motion abnormalities was suggestive of TCM. He was initiated on aspirin, atorvastatin, carvedilol, Lasix®, and Entresto^TM^ for coronary artery disease and heart failure with reduced EF. Scheduled oral liothyronine was added to his IV levothyroxine treatment. The patient was scheduled appointments with primary care, endocrine, and cardiology on discharge; However, the patient was thereafter lost to follow up.

## Discussion

The pathophysiology of TCM is multifactorial but partly stems from catecholamine excess during physical and emotional stress that causes myocardial dysfunction. TCM can manifest clinical findings similar to acute coronary syndrome, such as ST segment elevation with increased troponin and B-type natriuretic peptide levels. However, cardiac catheterization in TCM demonstrates the absence of obstructive coronary artery disease. Contributing mechanisms include direct toxicity of catecholamines on the myocardium, epicardial and microvascular coronary spasm, and increased cardiac workload [1]. 

Thyroid hormone homeostasis plays a key role within the interplay of catecholamines and TCM as T3/T4 sensitize cardiac adrenoreceptors to catecholamines through increased expression of beta-adrenoceptors on cardiac cells [2]. Under physiologic levels, this leads to increased chronotropy and inotropy. Interestingly, observational studies suggest that most cases of TCM in hypothyroid patients occur during thyroid replacement [3]. We postulate that the rapid replacement of T3 and T4 may increase the risk for TCM development via excessive potentiation of the catecholamine effect on cardiac adrenoreceptors. Furthermore, although TCM is often associated with hyperthyroid states, studies suggest that 35% of TCM cases associated with thyroid disease were in a hypothyroid state [4]. The study also found that a majority of cases of TCM in hypothyroid patients were on thyroid replacement therapy.

## Conclusions

The combination of the physical stress of myxedema coma and subsequent replacement of thyroid hormone is a likely culprit in inducing the patient’s myocardial dysfunction, highlighting the importance of maintaining clinical suspicion of TCM when patients progress through treatment. Anticipation of complications may be critical for timely and adequate supportive care, followed by treatment of the inciting cause of the physical or emotional stressor. Further research is needed to understand the exact mechanisms of TCM in the setting of hypothyroidism and how to prevent further morbidity.
